# Using Dynamic Multi-Task Non-Negative Matrix Factorization to Detect the Evolution of User Preferences in Collaborative Filtering

**DOI:** 10.1371/journal.pone.0135090

**Published:** 2015-08-13

**Authors:** Bin Ju, Yuntao Qian, Minchao Ye, Rong Ni, Chenxi Zhu

**Affiliations:** 1 Institute of Artificial Intelligence, College of Computer Science, Zhejiang University, Hangzhou, Zhejiang, P.R. China; 2 Health Information Center of Zhejiang Province, Hangzhou, Zhejiang, P.R. China; Jiangnan University, CHINA

## Abstract

Predicting what items will be selected by a target user in the future is an important function for recommendation systems. Matrix factorization techniques have been shown to achieve good performance on temporal rating-type data, but little is known about temporal item selection data. In this paper, we developed a unified model that combines Multi-task Non-negative Matrix Factorization and Linear Dynamical Systems to capture the evolution of user preferences. Specifically, user and item features are projected into latent factor space by factoring co-occurrence matrices into a common basis item-factor matrix and multiple factor-user matrices. Moreover, we represented both within and between relationships of multiple factor-user matrices using a state transition matrix to capture the changes in user preferences over time. The experiments show that our proposed algorithm outperforms the other algorithms on two real datasets, which were extracted from Netflix movies and Last.fm music. Furthermore, our model provides a novel dynamic topic model for tracking the evolution of the behavior of a user over time.

## Introduction

Personalized recommender systems are widely used in e-commerce, such as by Amazon and Netflix, to identify interesting products for their customers. Recommendation systems are often based on *Collaborative Filtering* (CF) [1–3], which relies only on past user feedback data, e.g., users’ previous transactions or item ratings. One type of user feedback data used in the CF literature is the explicit form of user-ratings, e.g., a 1–5 score. In this scenario, one of the challenges of CF of rating-type data is how to predict the missing values of the user-item score matrix effectively and efficiently, which has been explored widely [4–7]. However, in real-world scenarios most feedback is not explicit but implicit. Implicit feedback is tracked automatically, such as by monitoring clicks, view times, purchases, and other user activity. A common type of behavior in implicit feedback is user and item co-occurrence data reflecting user preferences, which we denote as selection data. There are many application scenarios in which a user can select the same item more than once, and we denote the behaviors in these scenarios as dynamic selection data. In the latter scenario, the data are usually divided into time steps, and the data in different time steps reflect different user preferences. Considering the dynamic selection data, an urgent and interesting question emerges: given the past behavior of the users when selecting items, how can the items that will be selected by the target user in the next time period be predicted?

**Fig 1:**
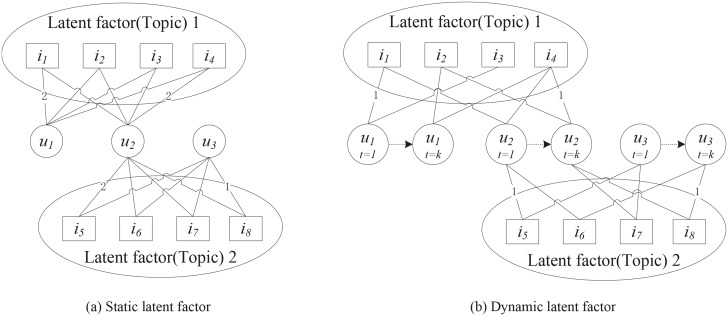
Topic Modeling in User Item Selection.

Tracking user preferences is a key issue in the task of prediction. A main approach to model user preference is to use latent factor models, e.g., latent semantic models [8–10] and matrix factorization models [4, 6], which learn a latent feature/factor vector for each user and each item in the dataset such that the inner product of these features minimizes an explicit or implicit cost function. This approach can also be considered as an example of co-clustering, where one cluster represents the item’s latent factor and the other represents the user’s latent factor. Fig 1(a) shows the result of performing topic modeling on user preferences without temporal considerations. The items selected by users *u*_1_, *u*_2_ and *u*_3_ are clustered according to topics 1 and 2 based on the weights (i.e., instances) of edges between users and items. The cluster of items is similar to the cluster of topic concepts. In fact, a user selects an item according to his preference, which is similar to words belonging to a document according to its topic. The topic concept clusters were derived using Probabilistic Latent Semantic Analysis (PLSA) [11] and Latent Dirichlet Allocation (LDA) [12]. These models exploit co-occurrence patterns of words in documents to unearth semantically meaningful probabilistic clusters of words. However, in practice, user preferences can change over time; therefore, this problem requires a method that is capable of modeling the temporal dependence of selections, i.e., a model that will allow us to provide the edge weights across time steps and preserve the topic distributions at different time steps. Such model could combine the temporal selections and construct dependencies between different time steps. See the scenario shown in Fig 1(b) as an example. We commonly refer to this type of models as temporal latent factor models.

In general, there are two types of temporal latent factor models for CF: temporal probabilistic topic models, such as the Dynamic Topic Model (DTM) [13], and dynamic latent factor-based matrix factorization approaches [5, 7, 14, 15]. However, both of these models have shortcomings. For example, DTM does not consider the evolution of user behavior, which is the main assumption in our work. Specifically, we assume that the topic-item distributions remain static over time but the preferences of the users change over time. Considering the dynamic changes in the preferences of users, Sahooet et al. proposed a temporal topic-user model [16], that extended from static latent factors representing users’ preferences in an Aspect Model [9] to dynamic latent factors with a Hidden Markov Model (HMM). Although this model considers dynamic changes in users’ preferences, it cannot predict the state of the user every time because it usually places most of the probability mass over a few states [16]. In contrast, the matrix factorization approaches have always been focused on predicting users’ ratings for items rather than users’ selection data. As noted above, in the case of temporal rating, the user can only rate an item once, but in reality, a user can select the same item many times as the user’s preference for the item changes over time. Therefore, these two types of models cannot be applied for modeling user item selection.

Fortunately, in recent years, Non-negative Matrix Factorization (NMF) has been extensively researched as a parts-based non-negative dictionary learning method [17]. Specifically, the non-negative constrain of NMF in latent factors space can be used to model a user’s preferences. In fact, many researchers have adopted NMF and its variants to solve CF [18–20]. Historically, PLSA and NMF were developed independently, but researchers later proved that PLSA solves the problem of NMF with KL I-divergence [21, 22]. To extend NMF to a dynamic latent factor model for selection data, Chua et al. [23] presented an algorithm called Dynamic Matrix Factorization (DMF), which combines NMF and Linear Dynamical Systems (LDS). However, DMF is not a unified model: it first uses NMF to generate an item-factor matrix as a global basis matrix for the observed data X* and then uses LDS to generate a state-transition matrix that governs the evolution of the factor-user matrices.

Motivated by DMF, we model dynamic selection data using a unified model that combines multi-task non-negative matrix factorization and a state transition matrix derived from LDS. Multi-task learning has become more popular because this method encourages learning tasks in parallel using a shared representation, which helps for each task learn better by using the other tasks’ information [24–26]. The multi-task NMF is a special case of non-negative tensor factorization (NTF) [27, 28], which is more flexible and useful in practice because it attempts to estimate only one common factor in the form of a basis matrix and some coefficient matrices over time [29]. In other words, the accumulative selection matrix X* is factorized into a common basis matrix for discovering item-factor and multiple factor-user matrices whose temporal relationships are governed by a transition matrix. The main contributions of this paper can be summarized as follows:

1. Our model provides a new method to embed common item factors and temporal user factors information into a unified model, in which user preference is tracked by a state transition matrix and every user has his/her own preference’s evolution profile.
2. Our work, which recovers the evolution of latent users’ factors can be interpreted as a special case of the dynamic topic model.
3. Compared with state-of-the-art methods, the proposed approach demonstrates superior performance in the prediction of future selection behavior.

The remainder of this paper is organized as follows: The ‘Analysis’ section introduces our model as well as the advantages and physical interpretations of the model. The multiplicative update algorithms for our model are also derived in this section. Experimental results from real world data are demonstrated and analyzed in the ‘Results’ section. Finally, conclusions are drawn in the ‘Discussion’ section.

## Analysis

### Dynamic Multi-task NMF for CF

Here, we formulate the problem by focusing on the dynamic selection of data. Given *K* time periods {*t*_1_, *t*_2_, …, *t*_*K*_}, in every time period, the users provide selection information about their preferences for known items, i.e., there are matrices ***X***_*k*_ = {***X***_1_, ***X***_2_, …, ***X***_*K*_} for *K* time periods. In each ***X***_*k*_, ***X***_*k*, *ij*_ = *x* represents a user *i* selecting *x* instances of an item *j* at time *k*. The accumulative selection matrix X* is obtained by X* = ∑_1:*K*_ ***X***_*k*_. The task of the time-sensitive recommender systems is to predict the items that a user will select in a future time period *K* + 1 given all of the selection matrices from the past *K* time periods.

In NMF, the goal is to find entrywise non-negative matrices ***W*** and ***H*** such that

$$\begin{array}{c} \left(W,H)=\underset{W,H}{\arg\min}𝓛(X∥WH\right) \end{array}$$ (1)

where the function 𝓛 is a suitable lost function and the ***X*** is the observation data matrix. The function 𝓛 can be Euclidian distance or divergence [17, 30]. In our model, the selection matrix $X\in R_{+}^{M\times N}$ is factorized into the item-factor matrix (or basis matrix) $W\in R_{+}^{M\times D}$ and the factor-user matrix (or coefficient matrix) $H\in R_{+}^{D\times N}$, where *M* is the number of items, *N* is the number of users, and *D* represents the size of dimension of the latent factors space. The item-factor matrix ***W*** represents the projection from items space to latent factors space, whereas the factor-user matrix ***H*** represents the coefficients for a user’s preferences for corresponding items [31]. We follow the classical assumption that the rows of the item-factor matrix $W_{i}^{T}$ and the columns of the factor-user matrix ***H***_*j*_ follow Gaussian prior distributions [4–6], as defined below:

$$\begin{array}{c} p(W_{i}^{T})=𝓝(W_{i}^{T}|\mu_{w},\sigma_{w}^{2}1),\;p(H_{j})=𝓝(H_{j}|\mu_{h},\sigma_{h}^{2}1). \end{array}$$ (2)

Although the observation data ***X***_*ij*_ are integers, the residuals $\left(X_{ij}-W_{i}^{T}H_{j}\right)$ should follow Gaussian distributions. Therefore, we define the conditional distribution over the observed data as

$$\begin{array}{c} p(X|W,H,\sigma^{2})=\prod_{i=1}^{N}\prod_{j=1}^{M}[𝓝(X_{ij}|W_{i}^{T}H_{j},\sigma^{2})]. \end{array}$$ (3)

where *σ* is the prior standard variance of the selection data.

**Fig 2:**
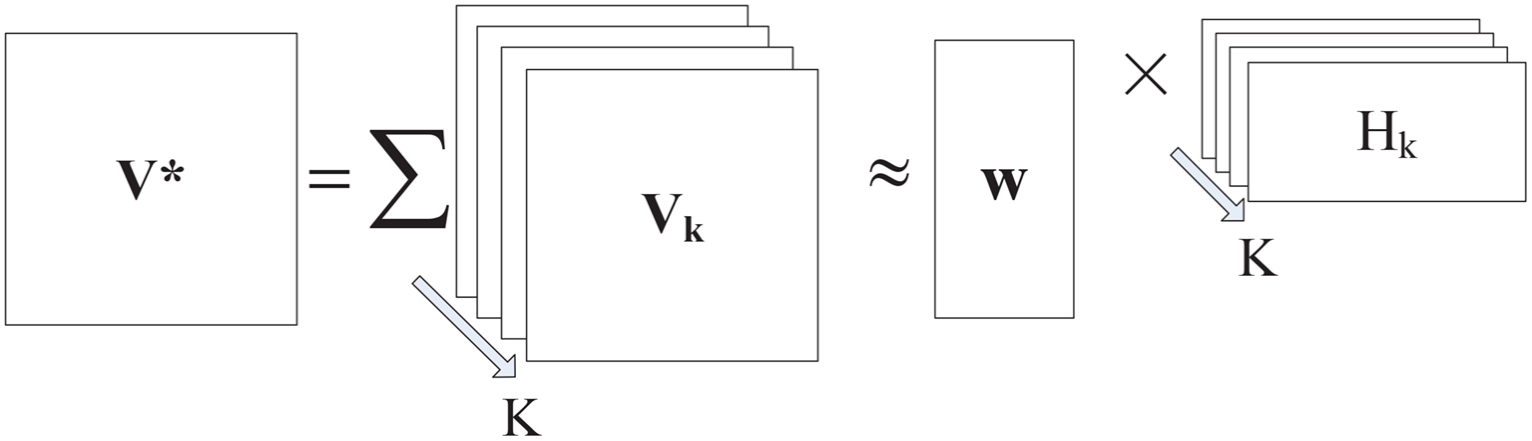
The framework of DMNMF.

**Fig 3:**
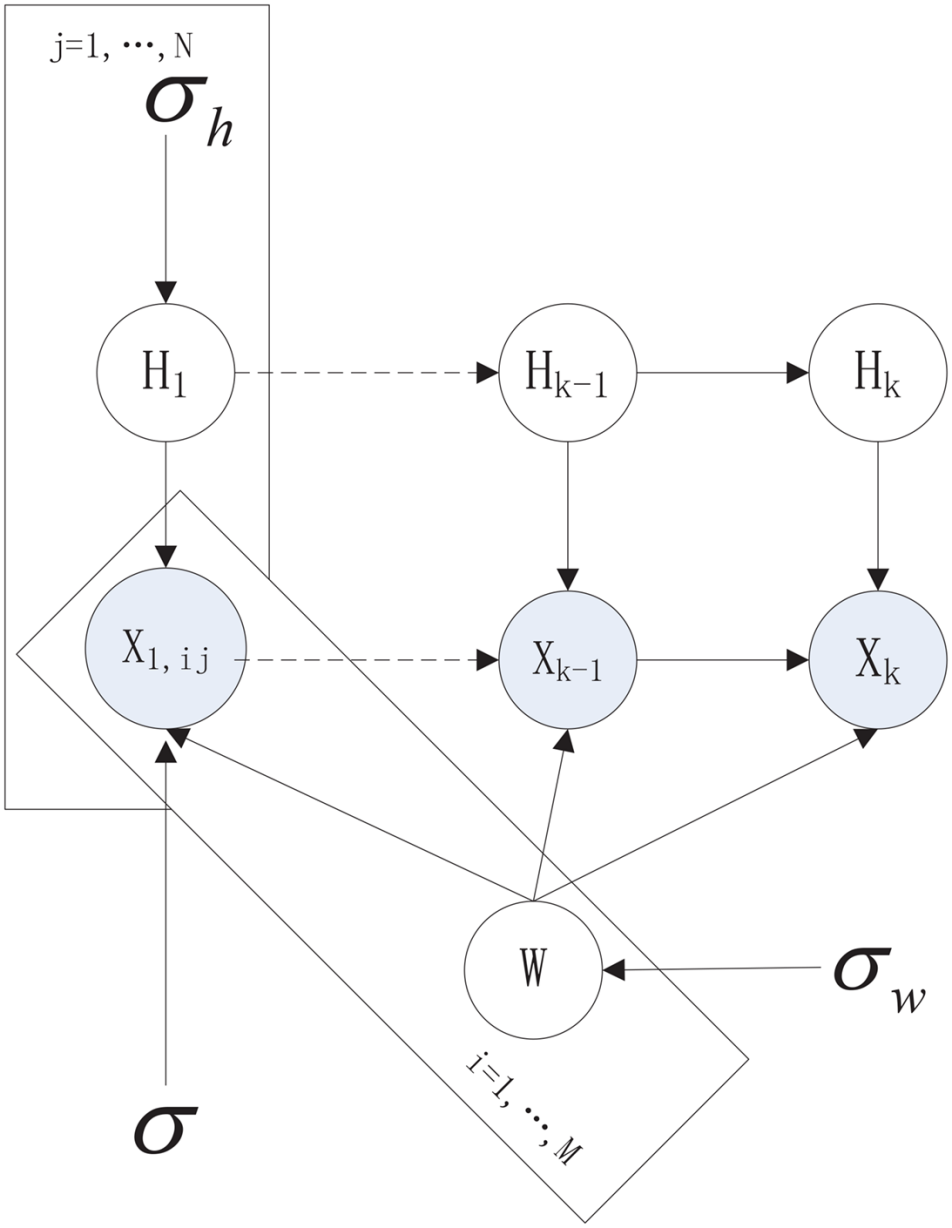
Probabilistic graphical model of DMNMF.

A direct and simple way to solve dynamic selection prediction is to perform NMF independently for each time step. However, this process reduces the predictive power because the lower ranking factors are not related across different time steps. In fact, we assume that item factors evolve very slowly and can be considered constant over time, whereas each user factors changes over time [32, 33], i.e., given *K* related input data matrices ***X***_1_, ***X***_2_, …, ***X***_*K*_, *K* coefficient matrices ***H***_1_, ***H***_2_, …, ***H***_*K*_ and a common basis matrix ***W*** can be factorized. According to the structure illustrated in Fig 2, we have a unified probabilistic graphical model, as shown in Fig 3.

As shown in Fig 3, a classical multi-task NMF model can be used. Multi-task NMF was first proposed in [34] for extracting common gene profiles. In our model, multi-task NMF can be seen as a combination of several strongly related NMF tasks which share the common item-factors matrix defined below:

$$\begin{array}{c} p(X_{k}|W,H_{k},\sigma^{2})=\prod_{k=1}^{K}\prod_{i=1}^{N}\prod_{j=1}^{M}[𝓝(X_{k,ij}|W_{i}^{T}H_{k,j},\sigma^{2})] \end{array}$$ (4)

where ***H***_*k*, *j*_ is the preference of user *j* during the *k* ^*th*^ time period.

Let us consider the relationship between ***H***_*k* − 1,*j*_ and ***H***_*k*, *j*_, which represent the changes in a user’s preferences from time period *k* − 1 to *k*. We adopt the idea of LDS [14, 23], which represents the mapping of latent factors from time step *k* − 1 to *k* using a state transition matrix ***A***_*k*_ with added Gaussian noise defined as follows:

$$\begin{array}{c} p(H_{k}|A_{k},H_{k-1},\sigma_{h}^{2})=\prod_{k=2}^{K}\prod_{i=1}^{D}\prod_{j=1}^{N}[𝓝(H_{k,ij}|A_{k,i}^{T}H_{k-1,j},\sigma_{h}^{2})] \end{array}$$ (5)

where *σ*_*h*_ is the prior standard variance of the factor-user latent variable and the matrix ***A***_*k*_ is different for each time period.

LDS cannot ensure the non-negativity of each *H*_*k*_, which is required by NMF. To avoid negativity, we propose the DMNMF model to combine multi-task NMF and LDS into a unified model using the log of the posterior distribution over the item-factor matrix, factor-user matrices and state transition matrix, as defined below:

$$\begin{array}{ccc} \ln p(W,H_{k},A|X,\sigma^{2},\sigma_{W}^{2},\sigma_{H}^{2}) & = & -\frac{1}{2\sigma^{2}}\sum_{k=1}^{K}\sum_{i=1}^{N}\sum_{j=1}^{M}{\left(X_{ij}-W_{i}^{T}H_{j}\right)}^{2}\\ & & -\frac{1}{2\sigma_{h}^{2}}\sum_{i=2}^{K}\sum_{i=1}^{D}\sum_{j=1}^{N}{\left(H_{k,ij}-A_{k,i}^{T}H_{k-1,j}\right)}^{2}+Const., \end{array}$$ (6)

where *Const*. is a constant that does not depend on the parameters. For the sake of simplicity, we fix ***A*** to avoid over-fitting and to capture interesting and important trends in the period. Maximizing the log-posterior with hyperparameters (i.e., the observation noise variance and prior variances) is equivalent to minimizing the sum-of-squared-errors cost function with quadratic regularization terms:

$$\begin{array}{ccc} C(\hat{W},\hat{A},{\hat{H}}_{k}) & = & \min_{W,H_{k},A}\frac{1}{2}(\sum_{k=1}^{K}{∥V_{k}-WH_{k}∥}_{F}^{2}+\lambda \sum_{k=2}^{K}{∥H_{k}-AH_{k-1}∥}_{F}^{2})\\ & & \text{s.t.}\;W\geq 0,A\geq 0,H_{k},\geq 0 \end{array}$$ (7)

where *C* is the cost function, $\lambda =\sigma^{2}/\sigma_{h}^{2}$, and ${\left\|.\right\|}_{F}^{2}$ denotes the Frobenius norm. The first item in the cost function represents the minimization of the errors between the observed data and the recovered data using the NMF *K* times. The second item represents the minimization of the errors that occur while estimating the transition matrix over *K* − 1 transitions.

Once the state transition matrix ***A*** is obtained, the coefficient matrix of the next time period ***H***_*K* + 1_ can be obtained by ***H***_*K* + 1_ = ***A*** ***H***_*K*_. Subsequently, we obtain the prediction function ***X***_*K* + 1, *ij*_ = *δ*(***W*** ***A*** ***H***_*K*_)_*i*, *j*_ = {1, (***W*** ***A*** ***H***_*K*_)_*i*, *j*_ > *τ*;0, otherwise.}, where *τ* is a threshold. Here, ***X***_*K* + 1, *ij*_ = 1 indicates that item *j* most likely would be selected by user *i* in the time period *K* + 1.

### Algorithm for DMNMF

Several algorithms have been proposed to solve the NMF problem, including multiplicative update (MU) [35], alternating least squares (ALS) [36], and projected gradient (PG) method [37], among others. Following [38], we adopt the MU algorithm to solve the optimization problem (7). To provide the MU algorithm for DMNMF, we first define the notations of two matrix operations ⊛ and ⊘, which represent element-wise multiplication and division, respectively. Let us consider the Karush-Kuhn-Tucker (KKT) conditions of (7), i.e.,

$$\begin{array}{c} \left\{ \begin{array}{ccc} W\geq 0 & & \left(8\right)\\ A\geq 0 & & \left(9\right)\\ H_{k}\geq 0, & k=1,\ldots ,K & \left(10\right)\\ \nabla_{W}C\geq 0 & & \left(11\right)\\ \nabla_{A}C\geq 0 & & \left(12\right)\\ \nabla_{H_{k}}C\geq 0, & k=1,\ldots ,K & \left(13\right)\\ W⊛\nabla_{W}C=0 & & \left(14\right)\\ A⊛\nabla_{A}C=0 & & \left(15\right)\\ H_{k}⊛\nabla_{H_{k}}C=0, & k=1,\ldots ,K & \left(16\right) \end{array}\right. \end{array}$$

where

$$\begin{array}{c} \nabla_{W}C=\sum_{k=1}^{K}(WH_{k}H_{k}^{T}-V_{k}H_{k}^{T}) \end{array}$$ (17)

and

$$\begin{array}{c} \nabla_{A}C=\sum_{k=2}^{K}(AH_{k-1}H_{k-1}^{T}-H_{k}H_{k-1}^{T}). \end{array}$$ (18)

Substituting (Eq 17) into (Eq 14), we have

$$\begin{array}{c} W⊛(\sum_{k=1}^{K}(WH_{k}H_{k}^{T}))=W⊛(\sum_{k=1}^{K}(V_{k}H_{k}^{T})). \end{array}$$ (19)

Then, the MU rule for ***W*** is derived, i.e.,

$$\begin{array}{c} W\leftarrow W⊛(\sum_{k=1}^{K}(V_{k}H_{k}^{T}))⊘(\sum_{k=1}^{K}(WH_{k}H_{k}^{T})). \end{array}$$ (20)

Likewise, we can obtain the MU rule for ***A*** via (Eq 15) and (Eq 18).

$$\begin{array}{c} A\leftarrow A⊛(\sum_{k=2}^{K}(H_{k}H_{k-1}^{T}))⊘(\sum_{k=2}^{K}(AH_{k-1}H_{k-1}^{T})). \end{array}$$ (21)

There are three derived cases for ***H***_*k*_. The first case is for *k* = 1,

$$\begin{array}{c} \nabla_{H_{1}}C=W^{T}(WH_{1}-V_{1})+\lambda A^{T}(AH_{1}-H_{2}) \end{array}$$ (22)

the second case for *k* ∈ [2, *K* − 1],

$$\begin{array}{c} \nabla_{\underset{2\leq k\leq K}{H_{k}}}C=W^{T}(WH_{k}-V_{k})+\lambda A^{T}(AH_{k}-H_{k+1})+\lambda (H_{k}-AH_{k-1}) \end{array}$$ (23)

and the third case is for *k* = *K*

$$\begin{array}{c} \nabla_{H_{K}}C=W^{T}(WH_{K}-V_{K})+\lambda (H_{K}-AH_{K-1}). \end{array}$$ (24)

Similarly, we can obtain the MU rule for ***H***_*k*_ via (Eq 16) and Eqs (22)(24) (23).

$$\begin{array}{c} H_{1}\leftarrow H_{1}⊛(W^{T}V_{1}+\lambda A^{T}H_{2})⊘(W^{T}WH_{1}+\lambda A^{T}AH_{1}) \end{array}$$ (25)

$$\begin{array}{c} H_{k}\leftarrow H_{k}⊛(W^{T}V_{k}+\lambda A^{T}H_{k+1}+\lambda AH_{k-1})⊘(W^{T}WH_{k}+\lambda A^{T}AH_{k}+\lambda H_{k}) \end{array}$$ (26)

$$\begin{array}{c} H_{K}\leftarrow H_{K}⊛(W^{T}V_{K}+\lambda AH_{K-1})⊘(W^{T}WH_{K}+\lambda H_{K}) \end{array}$$ (27)

**Theorem 1** *The cost function* *C* *in* (Eq 7) *is non-increasing under the update rules* (Eq 20) (Eq 21) *and* Eqs (25)–(27).

The proof is given in the appendix.

Based on the fact that *C* is non-increasing, the convergence of the MU algorithm is guaranteed. The detailed steps of the MU algorithm for the recommender are listed in Algorithm 1.

**Algorithm 1** The MU algorithm for DMNMF

**Input**:

  Data matrices to be factorized $V_{1},\ldots ,V_{K}\in R_{+}^{N\times M}$,

  Dimension size *D*,

  Regularization parameter *λ*,

  Threshold *τ*.

**Output**:

  Recommendation set ***X***.

1: Initialize ***W***, ***H***_1_, …, ***H***_*K*_ and ***A*** with random numbers in [0, 1].

2: **repeat**

3:    Fix ***H***_1_, …, ***H***_*K*_ and ***A***, update ***W*** with MU rule (Eq 20)

4:    Fix ***H***_1_, …, ***H***_*K*_ and ***W***, update ***A*** with MU rule (Eq 21)

5:    Fix ***W***, ***H***_2_, …, ***H***_*K*_ and ***A***, update ***H***_1_ with MU rule (Eq 25)

6:    Fix ***W***, ***H***_1_, …, ***H***_*K*−1_ and ***A***, update ***H***_*K*_ with MU rule (Eq 27)

7:    **for** *k* = 2 **to** *K*−1

8:     Fix ***W***, ***A***, ***H***_1_ and ***H***_*K*_, update ***H***_*k*_ with MU rule (Eq 26).

9:    **end for**

10: **until** the maximum number of iterations has been reached, or the change of cost function (7) in this iteration is less than a predefined threshold

Eq (20) costs *O*(*KMND*) time, Eq (21) costs *O*(*KD* ^2^ *N*) time, and Eq (26) costs *O*((*K*−2)(*MND*)) time. In summary, the total time complexity of Algorithm 1 is *O*(*TKMND*), where *T* is the number of iterations and is often set as a constant.

## Results

In this section, we validate the effectiveness of DMNMF by comparing with NMF, HMM-CF and DMF-IA using two datasets, i.e., Netflix [39] and Last.fm [40], obtained from real-world data. Below, we discuss how the training sets and test sets are constructed and how to measure the performances of the algorithms before reporting the results. Each algorithm is trained on data up to a certain time period *K*. These algorithm tasks of algorithms are then used to predict what each user will select in time period *K* + 1.

### Data Set

*Netflix Dataset*. Netflix has made available a dataset containing over 100 million ratings, containing 17,770 movies and approximately 480,000 users. The dataset consists of users’ ratings for movies along with the timestamp of the rating and spans 86 months. Using this dataset, we predict which movies a target user will rate in a given test period. We define a month as the time period and select 24 months as the time span of the dataset, which divides the temporal training sets into 23 matrices and uses the 24^*th*^ month as the test set. For a test set, we consider predicting which movies the user will rate as predicting which movies he/she will watch. To keep the training dataset size manageable, we construct matrices ***V*** ^*train*^ by reading the first 10,000 movie record files and selecting users who have rated at least 500 movies. The process for creating the training dataset is outlined as follows,

$$\begin{array}{c} \left\{\forall {\text{user}}_{j}:(\sum_{k=1}^{23}\sum_{i=1}^{10000}V_{i,j}^{train(k)}\geq 500)\right\} \end{array}$$ (28)

which results in a dataset with 1,015 users and 10,000 movies.

*Last.fm Dataset*. Last.fm is an Internet-based personalized radio station and music recommendation system. When the users of the service listen to music through a supported music player, Last.fm collects their music listening behavior. The data is used by Last.fm to make personalized music recommendations for their online radio station. This dataset contains time stamped records of users’ music listening activity. It has 992 users and 177,000 artists. The process of constructing the Last.fm training and test datasets is similar to that of Netflix.

We denote the sparsity of the dataset using the formula $sparsity=\frac{\sum_{i=1}^{M}\left(Item_{i}\right)}{N\times M}$, where *N* is the total number of users, *M* is the total number of items and *Item*_*i*_ is the number of item *i* that is selected by users. Summaries of the two training set of two datasets are shown on Table 1.

**Table 1:** DATASET SUMMARY.

Dataset	#users	#unique items	sparsity	Time span
Netflix	1,015	10,000	99.6%	Jan.2004−Nov.2005
Last.fm	992	5,000	99.3%	Jan.2007−Nov.2008

### Comparison

In this experiment, the following methods are compared:

1. NMF: As a static algorithm, NMF is the baseline for experiments. We feed NMF using an accumulated matrix X*, which sums up the frequencies of user-selected items from all time. After matrix completion by multiplying the two factorized matrices, the top-n sorted elements in the reconstructive matrix ***X*** with values greater than zero are recommended.
2. HMM-CF: The estimated HMM-CF with data observed up to *k* can be used to compute the latent class distribution for each user in time period *k* + 1 and then compute the distribution over the observation of articles in time period *k* + 1. The probability that the item *i* will be observed in *k* + 1 can be computed as $P\left(i\in I_{u}^{k+1}\right)=\sum_{d=1}^{D}P\left(Z_{u}^{k+1}=d\right)P\left(i\in I_{u}^{k+1};a_{d},b_{d},{\text{θ}}_{d}\right)$. Then, the items that are most likely to be observed in period *k* + 1 can be recommended to the user [16]. HMM-CF assumes that the distribution of how many items will be selected by a certain user in a month is a specific negative binomial distributions (NBD); therefore, the recommended number taken from the NBD for each user is a parameter, that is arbitrarily set by selecting each user’s top 5 or top 10 highest scoring items to recommended.
3. DMF-IA: According to the literature [23], DMF-IA is the variant of DMF that has the best performance. We first generate a basis matrix W using NMF; then, we use Kalman filtering to generate the fixed dynamic matrix ***A***. Finally, the top-n sorted elements in the reconstructive matrix ***X***, ***X*** = ***W*** ***A*** ***V***_*K*_ with values greater than zero are recommended.
4. DMNMF: We run the DMNMF algorithm as described above. In DMNMF, the sorted elements in the reconstructive matrix ***X*** = ***W*** ***A*** ***H***_*K*_ with values are greater than the threshold are recommended.

The performances of all of the algorithms are measured by their precision and recall scores. Only the items that user *i* actually selected in the time period *K* + 1 are considered correct recommendations. The precision, *P*, of the algorithm is the fraction of the recommended set that is correct and is defined as below:

$$\begin{array}{c} P=\frac{\sum_{i=1}^{L}hitItems_{i}}{\sum_{i=1}^{L}pItems_{i}}. \end{array}$$ (29)

where *L* is the number of all users who are in the recommended set, *pItems*_*i*_ refers to the number of items recommended to user *i*, *hitItems*_*i*_ refers to the number of items that are actually selected from the recommended set. The recall, *R*, of the algorithm is the fraction of the correct set that is recommended and is defined as below:

$$\begin{array}{c} R=\frac{\sum_{i=1}^{L}hitItems_{i}}{\sum_{i=1}^{B}bItems_{i}} \end{array}$$ (30)

where *B* is the number of all users who in the correct set and *bItems*_*i*_ refers to the number of items that the user *i* actually selected from the correct set. If more items are recommended, the precision will decrease, but the recall will increase. The harmonic mean is called the *F*1 score, see (Eq 31). The higher the *F*1 score is, the better the prediction performance is [41].

$$\begin{array}{c} F1=\frac{2\times P\times R}{P+R} \end{array}$$ (31)

To reduce the effect of randomness, we repeat these trials 500 times and compare the algorithms based on their average performances, as shown in Table 2. DMNMF significantly outperforms the other algorithms mainly because it captures the temporal changes in user preference in the unified model.

**Table 2:** PERFORMANCE COMPARISON AMONG DIFFERENT ALGORITHMS.

Algorithms	Netflix				Last.fm			
	*P*	*R*	*F*1	*AUC* *	*P*	*R*	*F*1	*AUC* *
NMF	0.1277	0.0370	0.0574	0.623[0.516,0.730]	0.1705	0.1467	0.1577	0.672[0.579,0.765]
HMM-CF	0.1264	0.0585	0.080	0.703[0.621,0,785]	**0.2919**	0.1573	0.2044	0.762[0.684,0.840]
DMF-IA	0.1118	0.0556	0.0743	0.677[0.577,0.777]	0.2109	0.1559	0.1793	0.780[0.700,0.86]
DMNMF	**0.1544**	**0.0834**	**0.1083**	**0.810[0.731, 0.831]**	0.2644	**0.1786**	**0.2312**	**0.896[0.871, 0.901]**

*:95% confidence intervals of AUC.

**Fig 4:**
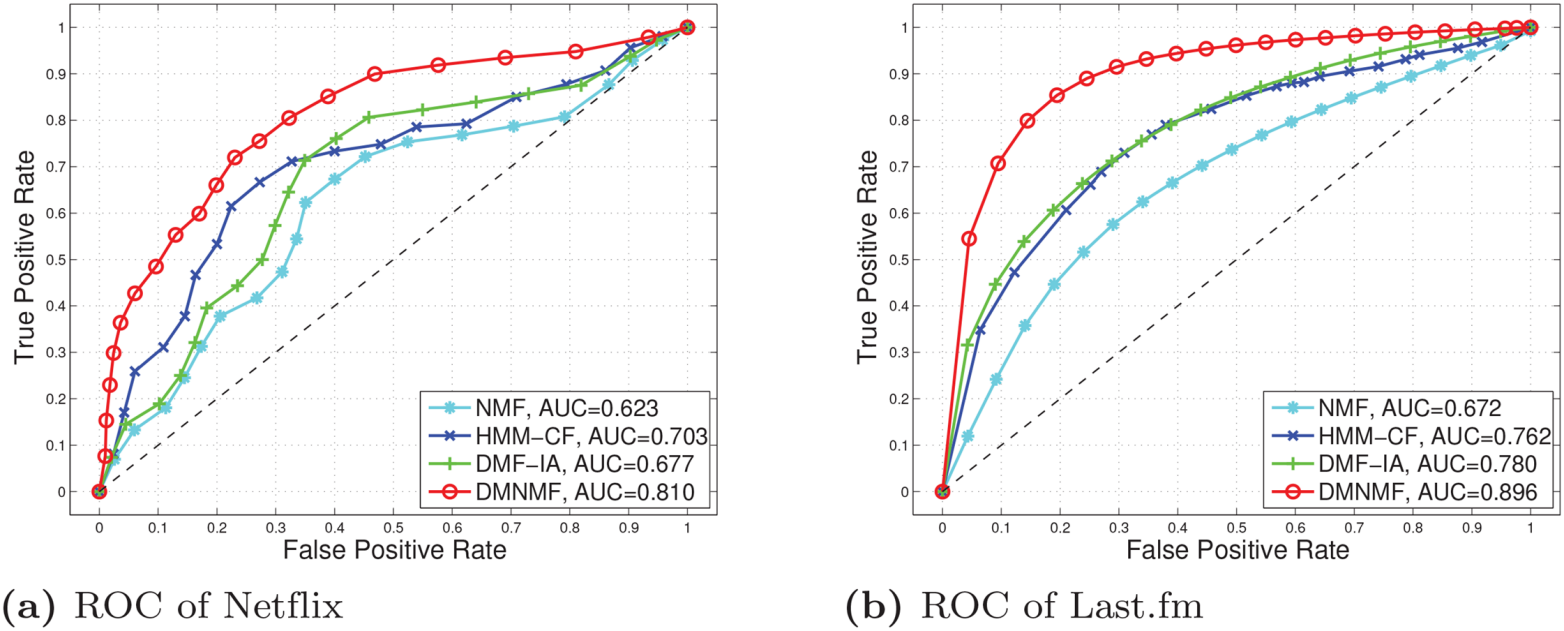
The ROC curves for evaluating the quality of the algorithms over the entire test datasets.

We are not only interested in precision and recall measures at a threshold or top-N quality of the recommended items but also the quality of the algorithms over the entire test dataset whose ranking score is produced by the algorithms. A receiver operating characteristic (ROC) curve is an intuitive way to compare multiple algorithms [42]. To draw an ROC curve, we split the sorted items score every 5 percentage and calculate the fraction of incorrect items recommended (false positive rate) and the fraction of correct items recommended (true positive rate). An ROC curve is a two-dimensional depiction of the prediction performance. We calculate the area-under-the-curve (AUC) to reduce the ROC performance to a single scalar value [43]. To generate confidence intervals for the AUC, we generate each ROC point 10 times and calculate the 95% confidence interval of the AUC using the method in [44]. According to Table 2, the average ROCs and the AUCs are shown in Fig 4. Fig 4 shows that DMNMF outperforms the other algorithms.

To obtain the runtime of these algorithms in Fig 4, when running the Netflix example, our new model takes 629s, which is comparable to HMM-CF (713s) and slightly slower than NMF (273s) and DMF-IA(395s).

### Parameter Learning for DMNMF

In our algorithm, there are three parameters: the latent factor sizes *D*, regularization parameter *λ* and threshold *τ* in the DMNMF model. To choose appropriate and robust parameters for the model, we propose a strategy to estimate a stable scope of parameters for the time-serial datasets. Similar to the idea of n-fold cross-validation, we split the original dataset into 12 time-continuous subsets for parameter validation. One subset contains 11 continuous month data as training sets and the following one month data as validation set. For example, for the first subset, the data set from the first month to the 11^*th*^ month are used as the training sets and the 12^*th*^ month is treated as the validation set, and for the second subset, the data set from the second to the 12^*th*^ month are used as the training sets ant the 13^*th*^ month is used as validation, and so on. As the original dataset is spanned of 24 months, we obtained 12 validation sets. The performances were then averaged over these validation sets.

**Fig 5:**
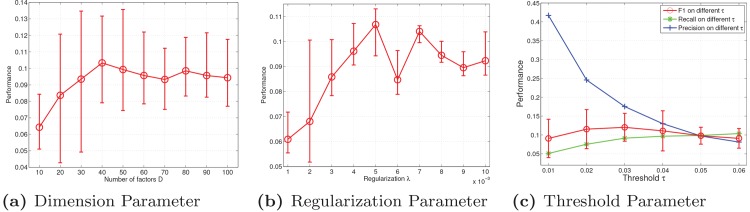
Performance values with different key parameters on Netflix.

For the sake of conciseness, we use the Netflix dataset as an example to demonstrate how the parameters were determined. Following the settings in the BPMF (Bayesian Probabilistic Matrix Factorization) model [7], which was run on the Netflix dataset, we set the *D* over the interval [10, 100] with step sizes of 10. As show in Fig 5(a), the predictive performance achieved the optimal performance when *D* = 40. As the number of latent factors increased, overfitting occurred.

The role of regulation *λ* is to balance the multi-task NMF term and LDS term in the cost function. Similar to [6], $\lambda =\sigma^{2}/\sigma_{h}^{2}$, indicating the ratio of the standard deviation of the observation data to the prior standard deviation of the factorized factor-user. Here, we choose *λ* from 0.001 to 0.01 with a step size of 0.001. As shown in Fig 5(b), *λ* is optimal at 0.005.

The threshold parameter *τ* controls the number of recommended items. The smaller the *τ* is, the larger the number of recommended items is. For example, when *τ* = 0.01, the number of predicting items is 202533 and the number of hitting items is 85813. Although the precision of the algorithm is 42.37%, each user is recommended over 200 items, which is called *over-recommended* in the recommender. Whereas when *τ* = 0.04, the number of predicting items is 22298 and the number of hitting items is 2898. Although the precision decreases substantially, only 22 items on average are recommended to each user, which results in the recommender providing a better user experience. We choose *τ* from 0.01 to 0.1 with a step size of 0.01. As shown in Fig 5(c), the threshold is optimal at 0.03. The confidence intervals of the precision and recall are omitted from Fig 5(c) for the sake of clarity.

**Fig 6:**
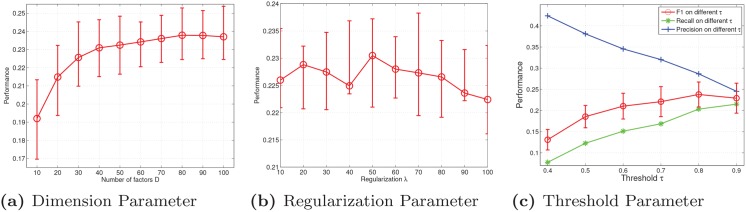
Performance values with different key parameters on Last.fm.

The parameter learning for the Last.fm dataset is very similar to that of the Netflix dataset, which is shown in Fig 6.

**Fig 7:**
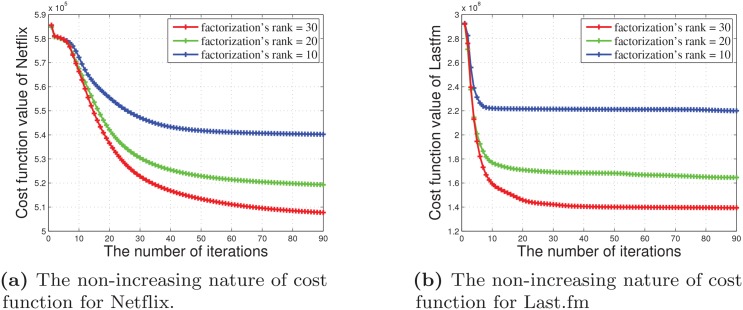
The non-increasing nature of cost function at various ranks.

We investigate the convergence of our DMNMF. Fig 7 shows the convergence curve of DMNMF and its cost function. The values of the cost function *C* with factorization ranks of 10, 20 and 30 were plotted. As shown in Fig 7, the non-increasing nature of *C* is obvious, and it drops very fast after a few iterations. However, the DMNMF model cannot ensure that the global minimum of the cost function is obtained. Therefore, there may be multiple local minimums, which depends on the initial points. Nevertheless, our numerical experiments showed that different initial values generate very similar results, which implies that the initial value might have only a small impact on the performance of the algorithm. Because the DMNMF cannot ensure that the global minimum is obtained when starting from a random initial condition, we examine the effects of the initial points on the final optimal solution. We randomly chosen the initial points 500 times to obtain the optimal solution of the DMNMF model. After initializing ***W***, ***H***_1_, …, ***H***_*K*_ and ***A*** with random numbers in [0, 1], we obtain the optimal factored matrices corresponding to the minimum value of the object function *C*.

### Case Study

**Fig 8:**
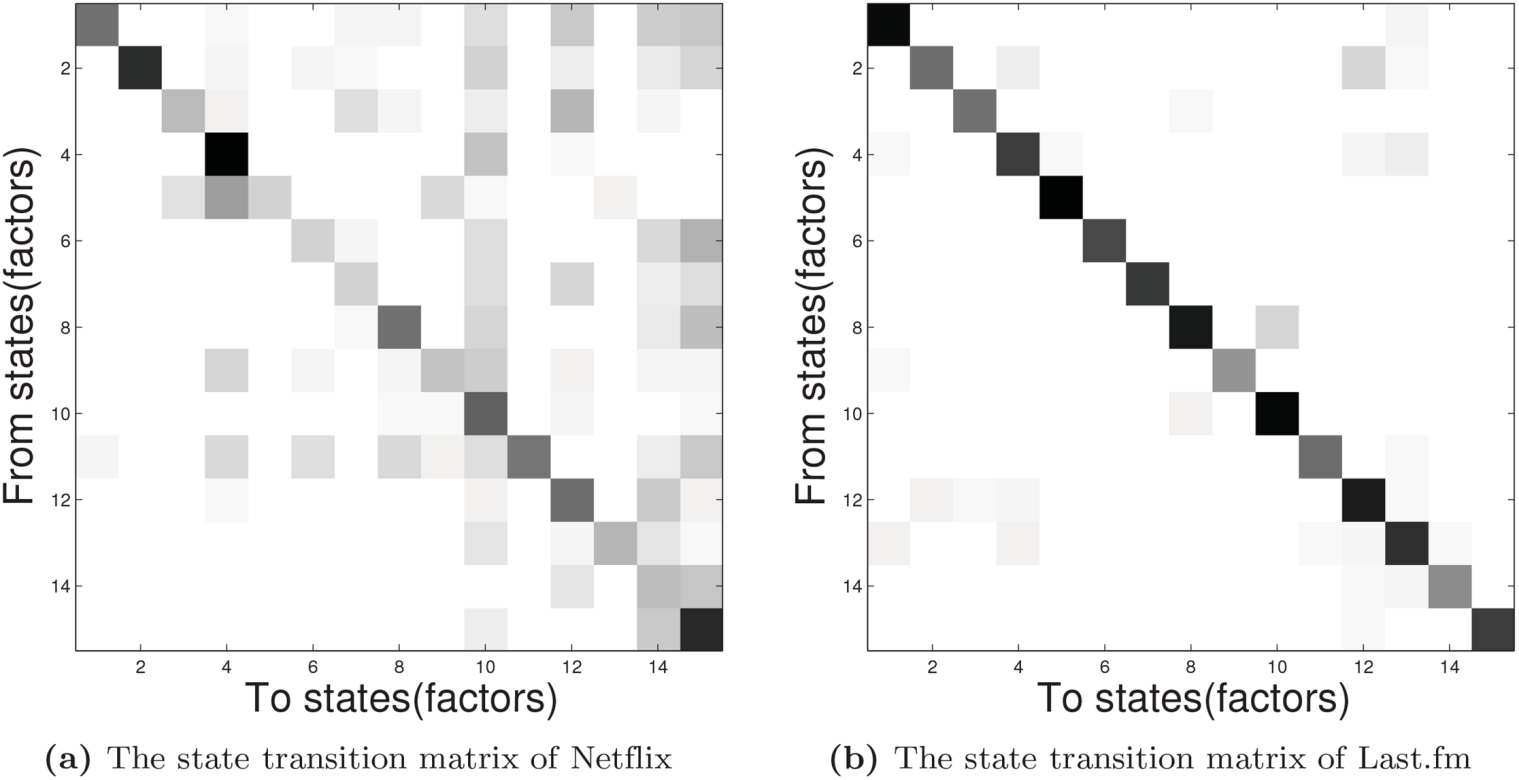
The graph of state transition matrix.

**Fig 9:**
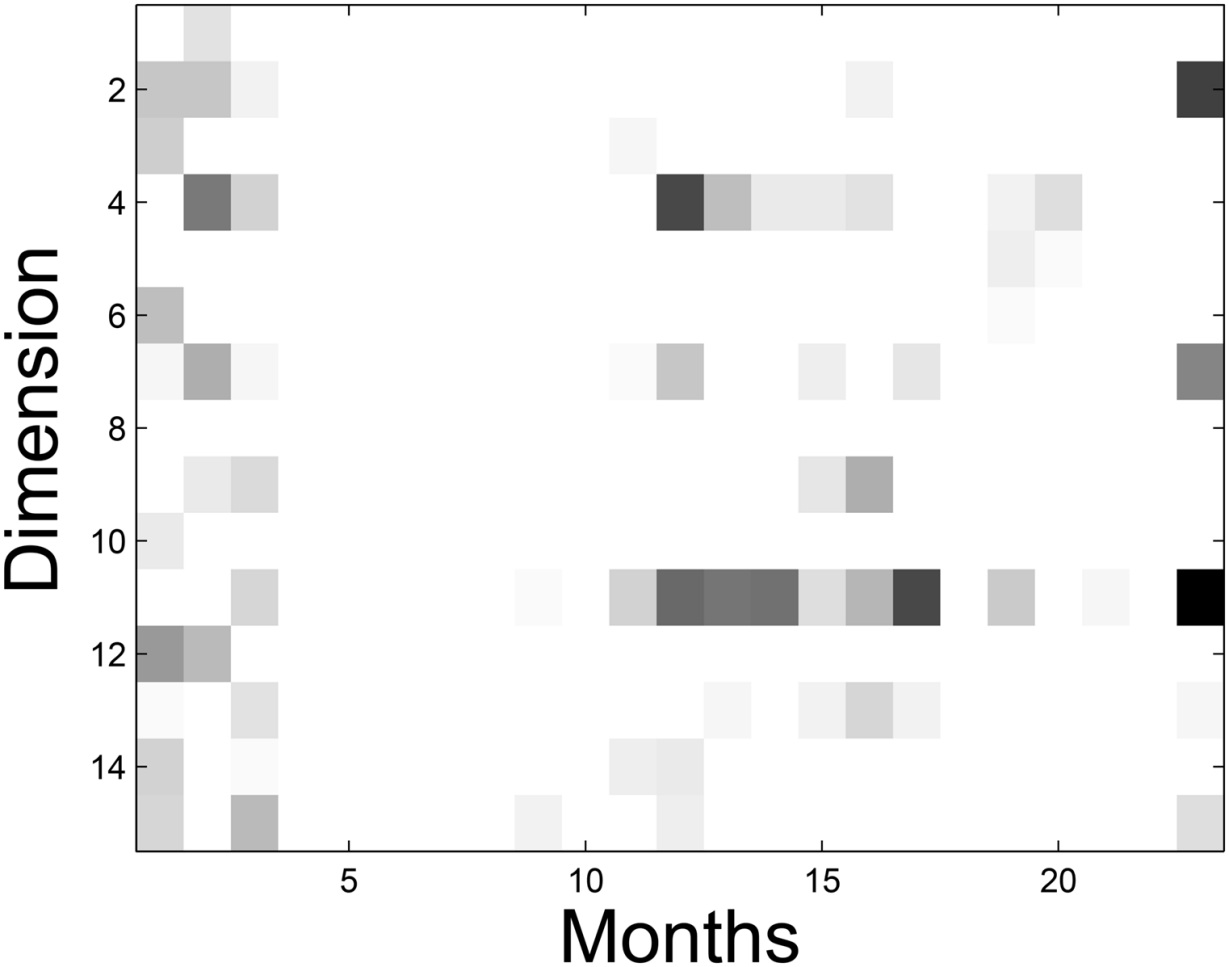
John’s preference profile.

A main feature of the DMNMF formulation is the use of dynamic matrix ***A*** to capture the evolution of a user’s latent factors *h*_*i*, *k*_ from one time step to the next time step. The latent state at *k* is given by *h*_*i*, *k*_ = ***A*** *h*_*i*, *k*−1_. The *k* ^*th*^ factor in *h*_*i*, *k*_ is derived from the dot product of the *k* ^*th*^ row of ***A*** and *h*_*i*, *k*−1_. The largest value in the *k* ^*th*^ row of ***A*** plays an important role in accounting for the value of the *k* ^*th*^ latent factor in *h*_*i*, *k*_. The state transition matrix of the two real datasets is shown in Fig 8. In Fig 8, the darker the state, the larger the value of the element in ***A***. To further illustrate the effectiveness of our DMNMF model, a case study is demonstrated using the Netflix dataset. For the sake of the clear visualization of the state transition matrix, we choose a dimension size of 15. We randomly select a user whose ID is 1371451(we name him ‘John’) and explain the evolution of his latent factors from the first month to the 23^*th*^ month (Jan. 2004 to Nov. 2005). We extract a certain column corresponding to John from every factor efficient ***H***_*k*_ and combine these into a matrix, then, we plot the matrix as an evolution of the preference profile that belongs to John. As shown in Fig 9, the larger the value of the element in the column, the darker the block and the more likely the corresponding preference factor. As time passes, the user’s changing preferences are represented by the largest element in the column. We find that John became active in selecting movies in the first 3 months and was dormant for a while. Then John became active in the 23^*rd*^ month, which is consistent with his behavior on rating movies in the training set.

We regard a factor as a ‘topics’ describing the user’s preference regarding certain properties of movies and select certain movies from corresponding topics that appear more black than others in Fig 9. For example, the top 10 largest elements (movies) are extracted from the corresponding column in the basis matrix ***W***, as shown in Table 3. Factor 2, factor 7, factor 11 can be interpreted as ‘Action’, ‘Romantic comedies’, ‘Family’, respectively. We demonstrate an example in which John shifted his preference from ‘Action’ to ‘Romantic comedies’ from Jan. 2004 to Nov. 2005. The ***W***_2,1_ entry in the 1^*st*^ column of the preference graph has the second highest value of 1.73, whereas the others have a mean value of 0.12; thus, we infer that John was interested in topic 2. Likewise, the ***W***_7,23_ entry in the 23^*rd*^ column of the preference graph has the highest value of 2.09, which indicates that John was interested in topic 7.

**Table 3:** LATENT TOPICS.

*Factor 2(Action)*	*Factor 7(Romantic comedies)*	*Factor 11(Family)*
Alien	The Object of My Affection	Curly Sue
I, Robot	Drive Me Crazy	Look Who’s Talking Too
Lord of the Rings: The Two Towers	Mickey Blue Eyes	Free Willy
Rambo: First Blood	Fools Rush In	Beethoven
Kill Bill	Down to You	Ray
Speed	Simply Irresistible	Finding Neverland
Star Trek	The Bachelor	The Forgotten
The Lost World: Jurassic Park	Green Card	Shark Tale
The Fifth Element	Mrs. Winterbourne	Napoleon Dynamite
X2: X-Men United	Picture Perfect	Junior

## Discussion

In collaborative filtering, item selection prediction is applied more widely than item rating prediction. This paper proposes an effective unified model called DMNMF to discover the latent factors behind users’ selection behaviors and capture the transition of user preference in latent factor space. We develop an MU algorithm to solve DMNMF. Experimental results on popular CF databases demonstrate that our proposed algorithm outperforms NMF, HMM and DMF as well as their extensions. As noted in the results section, our model has a larger time cost than the traditional NMF and DMF-IA models, mainly due to the longer time required for each iteration in our model compared with the others. Finally, our model can be used as a novel form of dynamic topic models for tracking the evolution of user preferences over time. In the future, we will develop a hybrid method that integrates the tracking of evolution of user preferences and the similarities between user preferences.

## Appendix

Proof of Multiplicative Update Rules (Eq 25)–(Eq 27)

As the cost function (7) is separable in the columns of ***H***_*k*_, we focus on one column of ***H***_*k*_ alone, which is denoted by ***h***_(*k*)_. To prove the non-increasing property of (Eq 26), an auxiliary function $G(h_{\left(k\right)},h_{\left(k\right)}^{t})$ is defined.

**Lemma 1** *The objective function* *C* *does not increase under the following update rule*

$$\begin{array}{c} h_{\left(k\right)}^{t+1}=\underset{h_{\left(k\right)}}{\arg\min}G\left(h_{\left(k\right)},h_{\left(k\right)}^{t}\right) \end{array}$$ (32)

*where* $G(h_{\left(k\right)},h_{\left(k\right)}^{t})$ *is an auxiliary function satisfying* *G*(***h***_(*k*)_, ***h***_(*k*)_) = *C*(***h***_(*k*)_) *and* $G(h_{\left(k\right)},h_{\left(k\right)}^{t})\geq C(h_{\left(k\right)})$.

This was proven in [35].

**Lemma 2** *If* $Q(h_{\left(k\right)}^{t})$ *is a diagonal matrix*

$$\begin{array}{c} Q_{ab}\left(h_{\left(k\right)}^{t}\right)=\delta_{ab}{\left((W^{T}W+\lambda A^{T}A+\lambda I)h_{\left(k\right)}^{t}\right)}_{a}/h_{(k),a}^{t} \end{array}$$ (33)

*then*

$$\begin{array}{ccc} G(h_{\left(k\right)}^{t},h_{\left(k\right)}) & = & C\left(h_{\left(k\right)}^{t}\right)+{\left(h_{\left(k\right)}-h_{\left(k\right)}^{t}\right)}^{T}\nabla_{h_{\left(k\right)}}C\left(h_{\left(k\right)}^{t}\right)+\\ & & \frac{1}{2}{\left(h_{\left(k\right)}-h_{\left(k\right)}^{t}\right)}^{T}Q\left(h_{\left(k\right)}^{t}\right)(h_{\left(k\right)}-h_{\left(k\right)}^{t}) \end{array}$$ (34)

*is an auxiliary function for* (7).

**Proof**: Since $G(h_{\left(k\right)},h_{\left(k\right)}^{t})=C(h_{\left(k\right)})$ is obviously, we need only show that $G(h_{\left(k\right)}^{t},h_{\left(k\right)})\geq C(h_{\left(k\right)})$. According to Taylor expansion,

$$\begin{array}{ccc} C(h_{\left(k\right)}) & = & C\left(h_{\left(k\right)}^{t}\right)+{\left(h_{\left(k\right)}-h_{\left(k\right)}^{t}\right)}^{T}\nabla_{h_{\left(k\right)}}C\left(h_{\left(k\right)}^{t}\right)+\\ & & \frac{1}{2}{\left(h_{\left(k\right)}-h_{\left(k\right)}^{t}\right)}^{T}(W^{T}W+\lambda A^{T}A+\lambda I)(h_{\left(k\right)}-h_{\left(k\right)}^{t}) \end{array}$$ (35)

we only has to proof

$$\begin{array}{c} G\left(h_{\left(k\right)}^{t},h_{\left(k\right)}\right)-C\left(h_{\left(k\right)}\right)=\frac{1}{2}{\left(h_{\left(k\right)}-h_{\left(k\right)}^{t}\right)}^{T}S(h_{\left(k\right)}-h_{\left(k\right)}^{t})\geq 0 \end{array}$$ (36)

where

$$\begin{array}{c} S=Q\left(h_{\left(k\right)}^{t}\right)-(W^{T}W+\lambda A^{T}A+\lambda I) \end{array}$$ (37)

To prove ***S*** is semipositive definite, consider the matrix:

$$\begin{array}{c} M_{ab}(h_{\left(k\right)}^{t})=h_{(k),a}^{t}{\left(S\right)}_{ab}h_{(k),b}^{t} \end{array}$$ (38)

which is just a rescaling of the components of ***S***. Then ***S*** is semipositive definite if and only if ***M*** is, and we denote

$$\begin{array}{c} Z=W^{T}W+\lambda A^{T}A+\lambda I \end{array}$$ (39)

then

$$\begin{array}{ccc} v^{T}Mv & = & \sum_{ab}v_{a}M_{ab}v_{b}\\ & = & \sum_{ab}h_{(k),a}^{t}{\left(Z\right)}_{ab}h_{(k),b}^{t}v_{a}^{2}-v_{a}h_{(k),a}^{t}{\left(Z\right)}_{ab}h_{(k),b}^{t}v_{b}\\ & = & \sum_{ab}{\left(Z\right)}_{ab}h_{(k),a}^{t}h_{(k),b}^{t}[\frac{1}{2}v_{a}^{2}+\frac{1}{2}v_{b}^{2}-v_{a}v_{b}]\\ & = & \frac{1}{2}\sum_{ab}{\left(Z\right)}_{ab}h_{(k),a}^{t}h_{(k),b}^{t}{\left(v_{a}-v_{b}\right)}^{2}\\ & \geq & 0 \end{array}$$ (40)

Therefore, $G(h_{\left(k\right)}^{t},h_{\left(k\right)})\geq C(h_{\left(k\right)})$ is proven according to (36).

**Proof**: [Proof of rules (25)–(27)] To minimize the auxiliary function *G*, we set

$$\begin{array}{c} \nabla_{h_{\left(k\right)}}G\left(h_{\left(k\right)}^{t},h_{\left(k\right)}\right)=\nabla_{h_{\left(k\right)}}C\left(h_{\left(k\right)}^{t}\right)+Q\left(h_{\left(k\right)}^{t}\right)(h_{\left(k\right)}-h_{\left(k\right)}^{t})=0 \end{array}$$ (41)

Solving ***h***_(*k*)_, we have

$$\begin{array}{ccc} h_{\left(k\right)} & = & h_{\left(k\right)}^{t}-Q^{-1}\left(h_{\left(k\right)}^{t}\right)\nabla_{h_{\left(k\right)}}C\left(h_{\left(k\right)}^{t}\right)\\ & = & h_{\left(k\right)}^{t}-\text{diag}(h_{\left(k\right)}^{t}⊘(W^{T}W+\lambda A^{T}A+\lambda I)h_{\left(k\right)}^{t})\nabla_{h_{\left(k\right)}}C\left(h_{\left(k\right)}^{t}\right)\\ & = & h_{\left(k\right)}^{t}-h_{\left(k\right)}^{t}⊘(W^{T}Wh_{\left(k\right)}^{t}+\lambda A^{T}Ah_{\left(k\right)}^{t}+\lambda h_{\left(k\right)}^{t})\\ & & \;⊛[W^{T}(Wh_{\left(k\right)}^{t}-V_{k})+\lambda A^{T}(Ah_{\left(k\right)}^{t}-h_{\left(k+1\right)})+\lambda (h_{\left(k\right)}^{t}-Ah_{\left(k-1\right)})]\\ & = & h_{\left(k\right)}^{t}⊛(W^{T}V_{k}+\lambda A^{T}h_{\left(k+1\right)}+\lambda Ah_{\left(k-1\right)})⊘(W^{T}Wh_{\left(k\right)}^{t}+\lambda A^{T}Ah_{\left(k\right)}^{t}+\lambda h_{\left(k\right)}^{t}) \end{array}$$ (42)

According to Lemma 1, objective function (7) is non-increasing under the update rule (26). Obviously, objective function (7) is non-increasing under the rules (25) and (27), because they are the special cases of the rule (26).

Proof of Multiplicative Update Rules (20) and (21)

Since the proof of update rule (20) is exactly the same as (21), we only present the proof of update rule (21). As the objective function (7) is separable in the columns of ***A***, we focus on one column of ***A*** alone, which is denoted by ***a***. To prove the non-increasing property of (21), an auxiliary function *G*(***a***, ***a*** ^*t*^) is defined.

**Lemma 3** *If* ***Q***(***a*** ^*t*^) *is a diagonal matrix*

$$\begin{array}{c} Q_{ij}\left(a^{t}\right)=\delta_{ij}(\sum_{k=2}^{K}({\left(a^{t}H_{k-1}H_{k-1}^{T}\right)}_{i}/a_{i}^{t})) \end{array}$$ (43)

*then*

$$\begin{array}{c} G\left(a,a^{t}\right)=C\left(a^{t}\right)+{\left(a-a^{t}\right)}^{T}\nabla_{a}C\left(a^{t}\right)+\frac{1}{2}{\left(a-a^{t}\right)}^{T}Q\left(a^{t}\right)(a-a^{t}) \end{array}$$ (44)

*is an auxiliary function for* (7).

**Proof**: *G*(***a***, ***a***) = *C*(***a***) obviously holds. According to Taylor expansion,

$$\begin{array}{ccc} C(a) & = & C\left(a^{t}\right)+{\left(a-a^{t}\right)}^{T}\nabla_{a}C\left(a^{t}\right)+\frac{1}{2}{\left(a-a^{t}\right)}^{T}(\sum_{k=2}^{K}(H_{k-1}H_{k-1}^{T}))(a-a^{t}) \end{array}$$ (45)

we have

$$\begin{array}{c} G\left(a,a^{t}\right)-C\left(a\right)=\frac{1}{2}(a-a^{t})S{\left(a-a^{t}\right)}^{T} \end{array}$$ (46)

where

$$\begin{array}{ccc} S & = & Q\left(a^{t}\right)-\sum_{k=2}^{K}(H_{k-1}H_{k-1}^{T})\\ & = & \sum_{k=2}^{K}(\text{diag}((aH_{k-1}H_{k-1}^{T})⊘a^{t})-H_{k-1}H_{k-1}^{T}) \end{array}$$ (47)

We denote

$$\begin{array}{c} M_{k}=\text{diag}((aH_{k-1}H_{k-1}^{T})⊘a^{t})-H_{k-1}H_{k-1}^{T} \end{array}$$ (48)

It was proven in [35] that each ***M***_*k*_ is positive semidefinite. Since the sum of positive semidefinite matrices is positive semidefinite, $S=\sum_{k=2}^{K}M_{k}$ is a positive semidefinite matrix. Therefore, *G*(***a***, ***a*** ^*t*^) ≥ *C*(***a***) is proven according to (46).

**Proof**: [Proof of rules (21)] To minimize the auxiliary function *G*, we set

$$\begin{array}{c} \nabla_{a}G\left(a,a^{t}\right)=\nabla_{a}C\left(a^{t}\right)+Q\left(a^{t}\right)(a-a^{t})=0 \end{array}$$ (49)

Solving ***a***, we have

$$\begin{array}{ccc} a & = & a^{t}-Q^{-1}\left(a^{t}\right)\nabla_{a}C\left(a^{t}\right)\\ & = & a^{t}-\text{diag}(a^{t}⊘\sum_{k=2}^{K}(a^{t}H_{k-1}H_{k-1}^{T}))\nabla_{a}C\left(a^{t}\right)\\ & = & a^{t}-a^{t}⊘\sum_{k=2}^{K}(a^{t}H_{k-1}H_{k-1}^{T})⊛\sum_{k=2}^{K}(a^{t}H_{k-1}H_{k-1}^{T}-H_{k-1}H_{k-1}^{T})\\ & = & a^{t}⊛(\sum_{k=2}^{K}(H_{k-1}H_{k-1}^{T}))⊘(\sum_{k=2}^{K}(a^{t}H_{k-1}H_{k-1}^{T})) \end{array}$$ (50)

According to Lemma 1, objective function (7) is non-increasing under the update rule (21).
