# Correction: Using Dynamic Multi-Task Non-Negative Matrix Factorization to Detect the Evolution of User Preferences in Collaborative Filtering

**DOI:** 10.1371/journal.pone.0138279

**Published:** 2015-09-14

**Authors:** Bin Ju, Yuntao Qian, Minchao Ye, Rong Ni, Chenxi Zhu

The affiliation for the corresponding author is incorrect. Yuntao Qian is not affiliated with #2 but with #1 Institute of Artificial Intelligence, College of Computer Science, Zhejiang University, Hangzhou, Zhejiang, P.R. China.
